# Congenital Pulmonary Artery Anomalies: What Every Radiologist Should Know

**DOI:** 10.5334/jbsr.4159

**Published:** 2026-02-09

**Authors:** Geewon Lee, Ji Won Lee

**Affiliations:** 1 Department of Radiology, Pusan National University Hospital, Pusan National University School of Medicine and Medical Research Institute, Busan, Republic of Korea

**Keywords:** pulmonary artery, congenital abnormalities, tomography, X‑ray computed

## Abstract

Congenital pulmonary artery anomalies may remain undetected until adulthood, and radiologists must recognize these anomalies to avoid misdiagnosis and guide clinical decisions. This pictorial essay reviews several congenital pulmonary artery anomalies and illustrates their representative imaging findings.

## Introduction

The pulmonary arteries are blood vessels transporting deoxygenated blood from the right ventricle (RV) to the lungs. Contrast‑enhanced CT angiography is used as a noninvasive tool for the diagnosis of pulmonary artery disease [1]. This pictorial essay illustrates the chest radiography and CT features of congenital anomalies of the pulmonary arteries.

### Pulmonary artery sling

**Figure 1:**
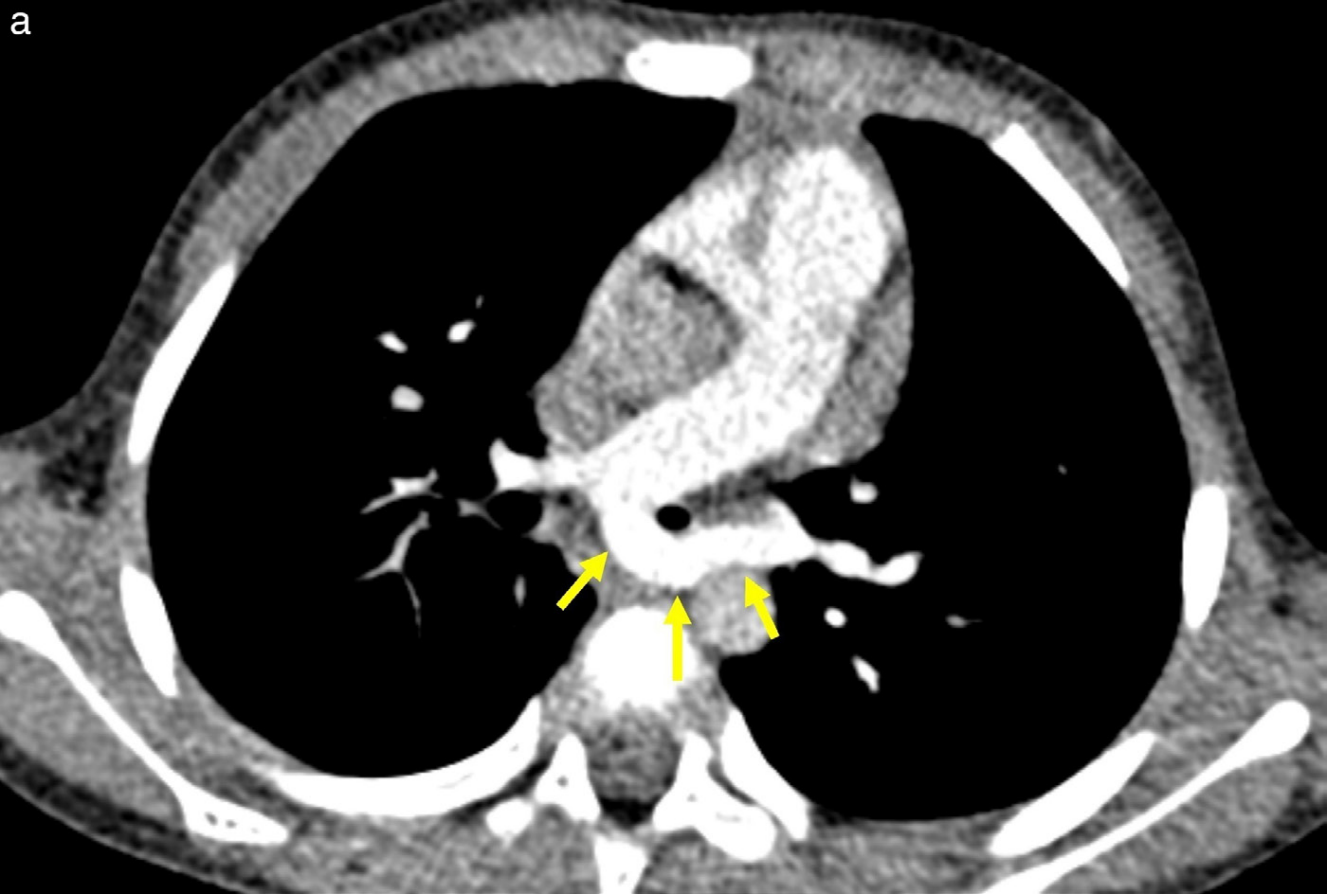

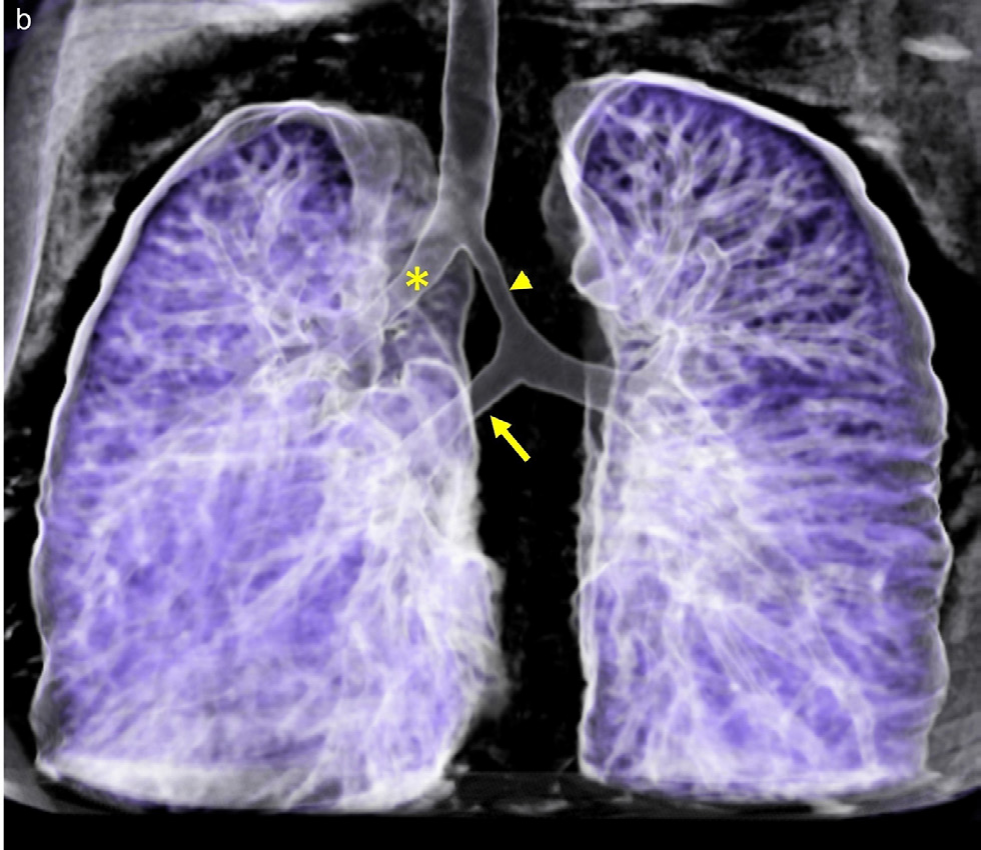

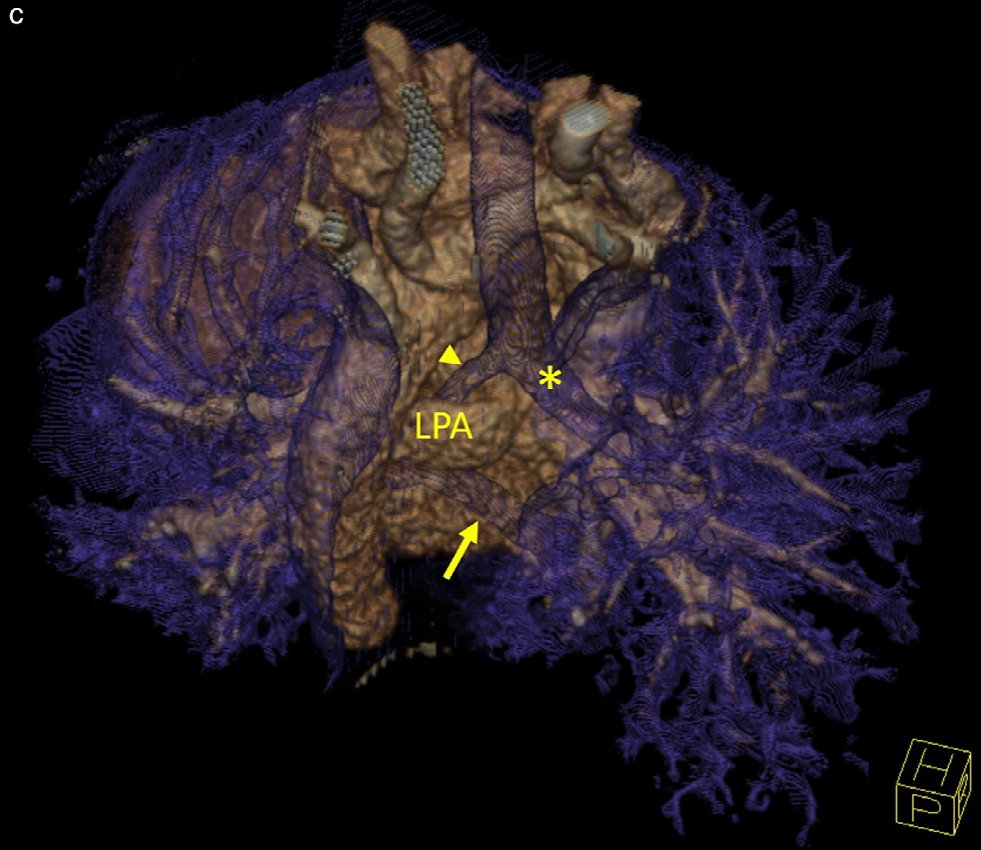
Pulmonary artery sling. **(a)** Axial CT image reveals the LPA (arrows) anomalously originating from the posterior aspect of RPA. **(b–c)** Volume‑rendered images in the anterior‑posterior **(b)** and posterior‑anterior **(c)** views illustrate a right upper lobe tracheal bronchus (asterisk) arising near the expected carina, severe distal tracheal narrowing (arrowhead), and an anomalous bridging bronchus (arrow).

**Figure 2:**
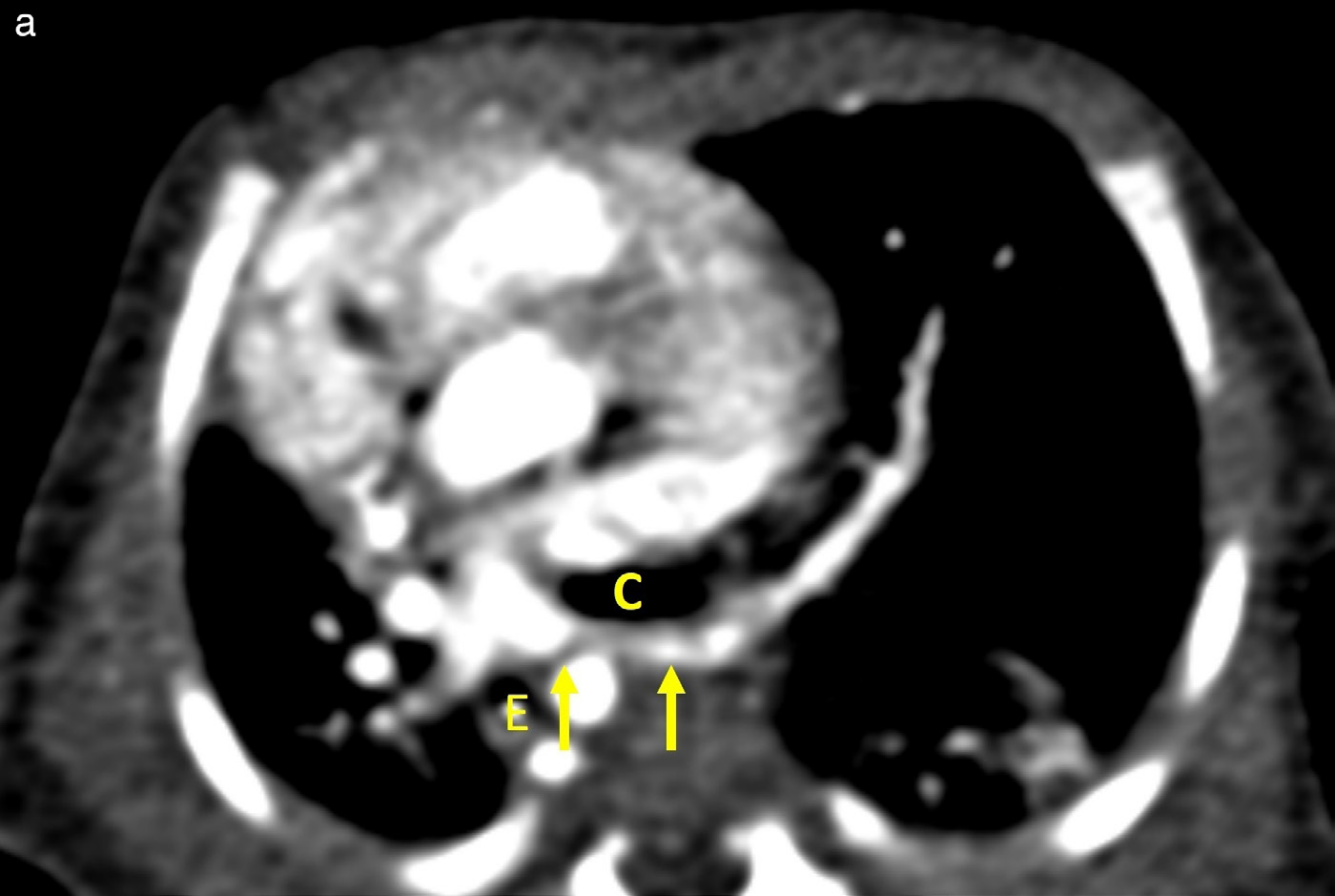

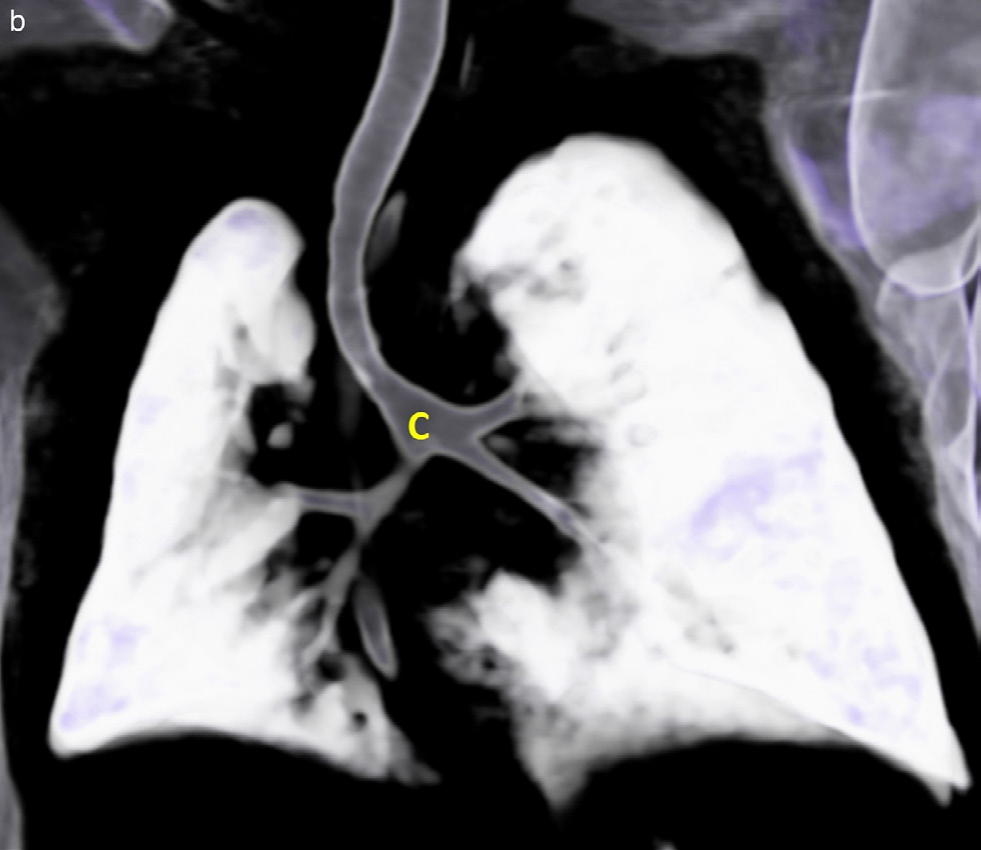

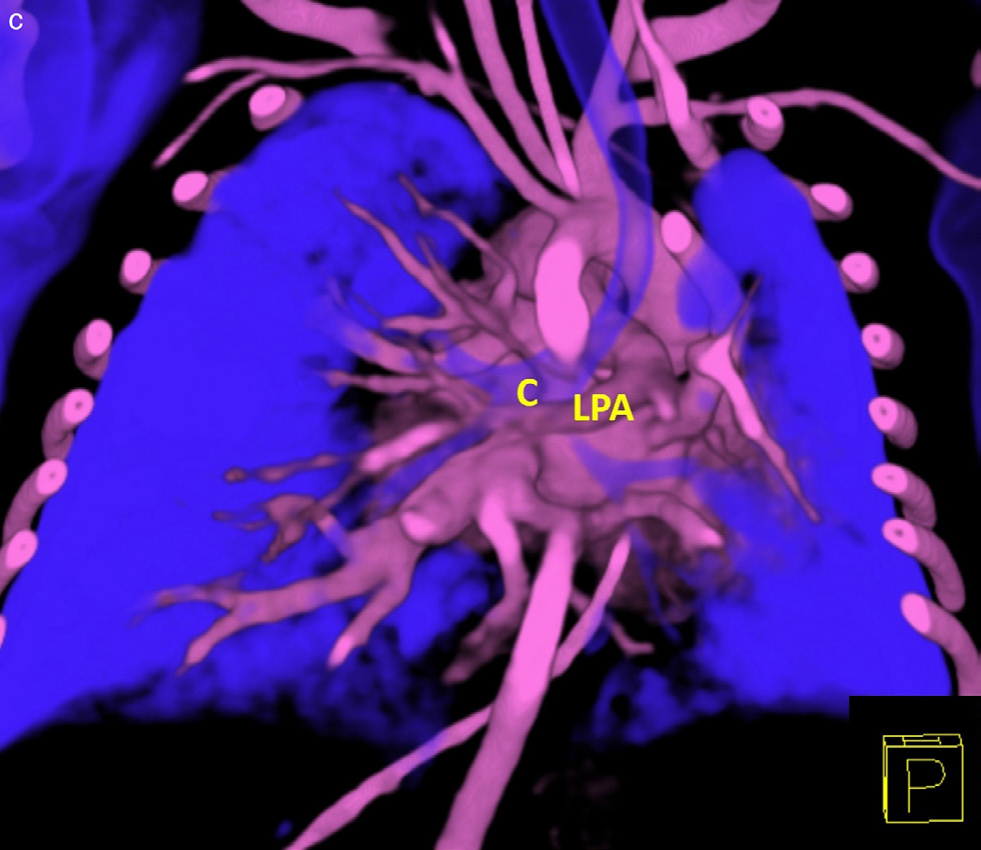
Pulmonary artery sling. **(a)** Axial CT image at the T6 level reveals an anomalous origin of the LPA (arrows) from the RPA. **(b–c)** Volume‑rendered images in the anterior‑posterior **(b)** and posterior‑anterior **(c)** views reveal a pulmonary artery sling located just above the low‑lying carina with no separate right upper lobe bronchus. C, Carina of the trachea; E, Esophagus.

In patients with a pulmonary artery sling (PAS), also referred to as an aberrant left pulmonary artery (ALPA), the LPA arises abnormally from the posterior aspect of the right pulmonary artery (RPA) [2]. PAS is classified into two types based on the level of LPA origin and the associated airway anatomy. Type I originates just above the carina (T4–T5), with or without a tracheal bronchus. In contrast, type II arises at a lower level (T5–T6) and is frequently associated with long‑segment tracheal stenosis and abnormal bronchial branching, such as a bridging bronchus (Figures 1 and 2) [2]. Barium esophagography may also show an anterior indentation of the esophagus in PAS patients [3].

Treatment depends on clinical symptoms and anatomy. Asymptomatic patients can be monitored clinically, whereas patients with respiratory symptoms require the reimplantation of the LPA to the left [2].

### Partial anomalous left pulmonary artery

**Figure 3:**
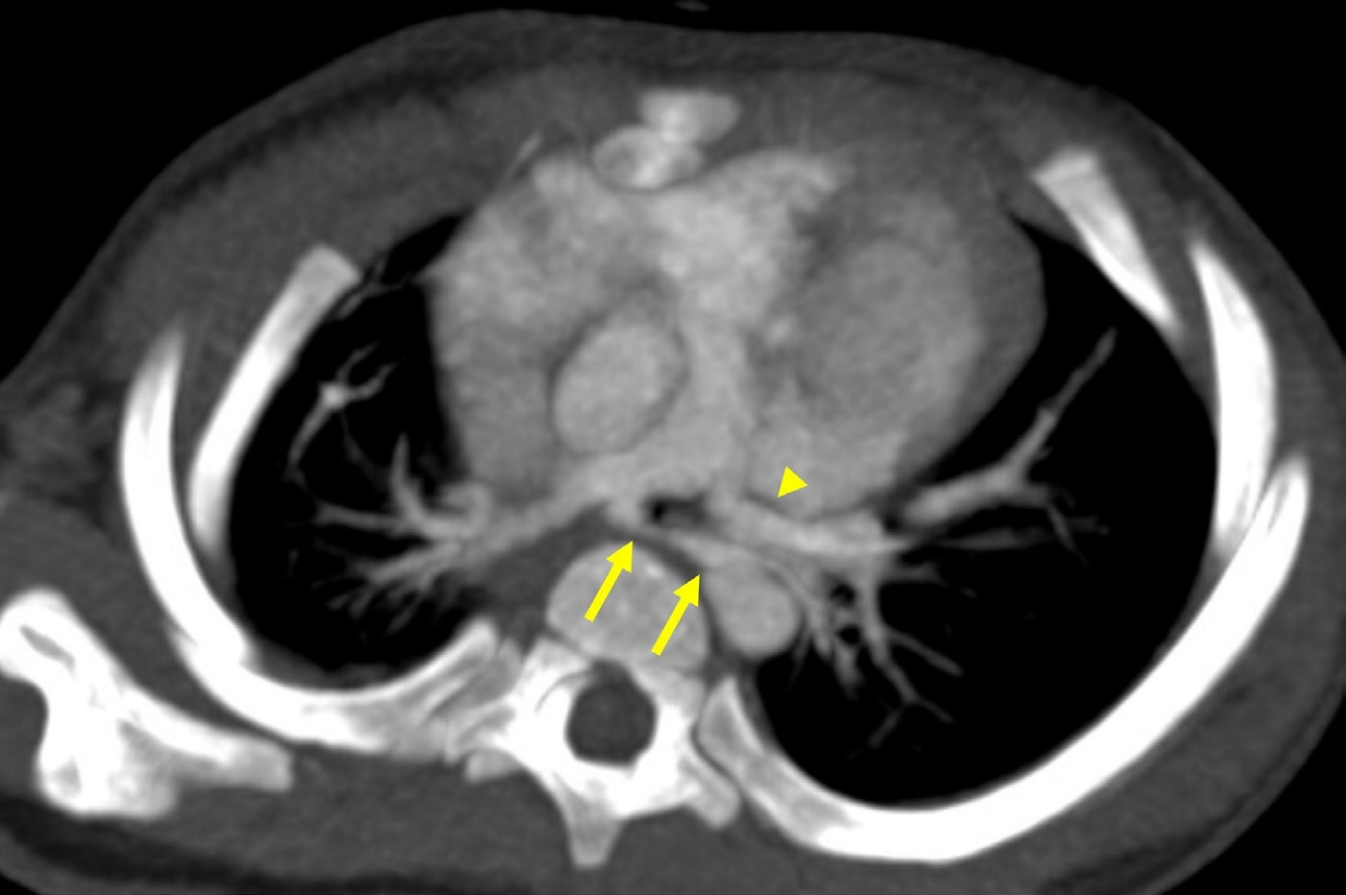
Partial anomalous left pulmonary artery. Axial maximum intensity projection image shows a posteriorly coursing partial anomalous left pulmonary artery (arrows) arising from the RPA. A normally originating LPA is also seen (arrowhead).

Partial anomalous left pulmonary artery, also referred to as duplicated LPA, is an exceptionally rare congenital vascular variant. It is defined by the presence of an arterial branch originating from the RPA and supplying the left lung, while a normally arising LPA is also present [4–6] (Figure 3). Most anomalous branches course toward the left lower lobe [5]. Partial anomalous left pulmonary arteries are classified by their position relative to the tracheobronchial tree: anterior, anteroinferior, or posterior. The posterior type forms a partial PAS, and this entity should be distinguished from a left PAS, in which the entire LPA arises aberrantly from the RPA [7, 8].

### Idiopathic dilatation of the pulmonary trunk

Idiopathic dilatation of the pulmonary trunk is a rare entity characterized by abnormal enlargement of the pulmonary trunk without abnormal cardiac or extracardiac shunts, cardiac disease, or pulmonary disease [9]. It is a diagnosis of exclusion, made after ruling out conditions that could dilate the pulmonary trunk, including pulmonary hypertension, connective tissue disorders, or cardiac disease (mainly pulmonary valve stenosis). Normal pressure in the RV and pulmonary artery must be confirmed using invasive angiography [10].

**Figure 4:**
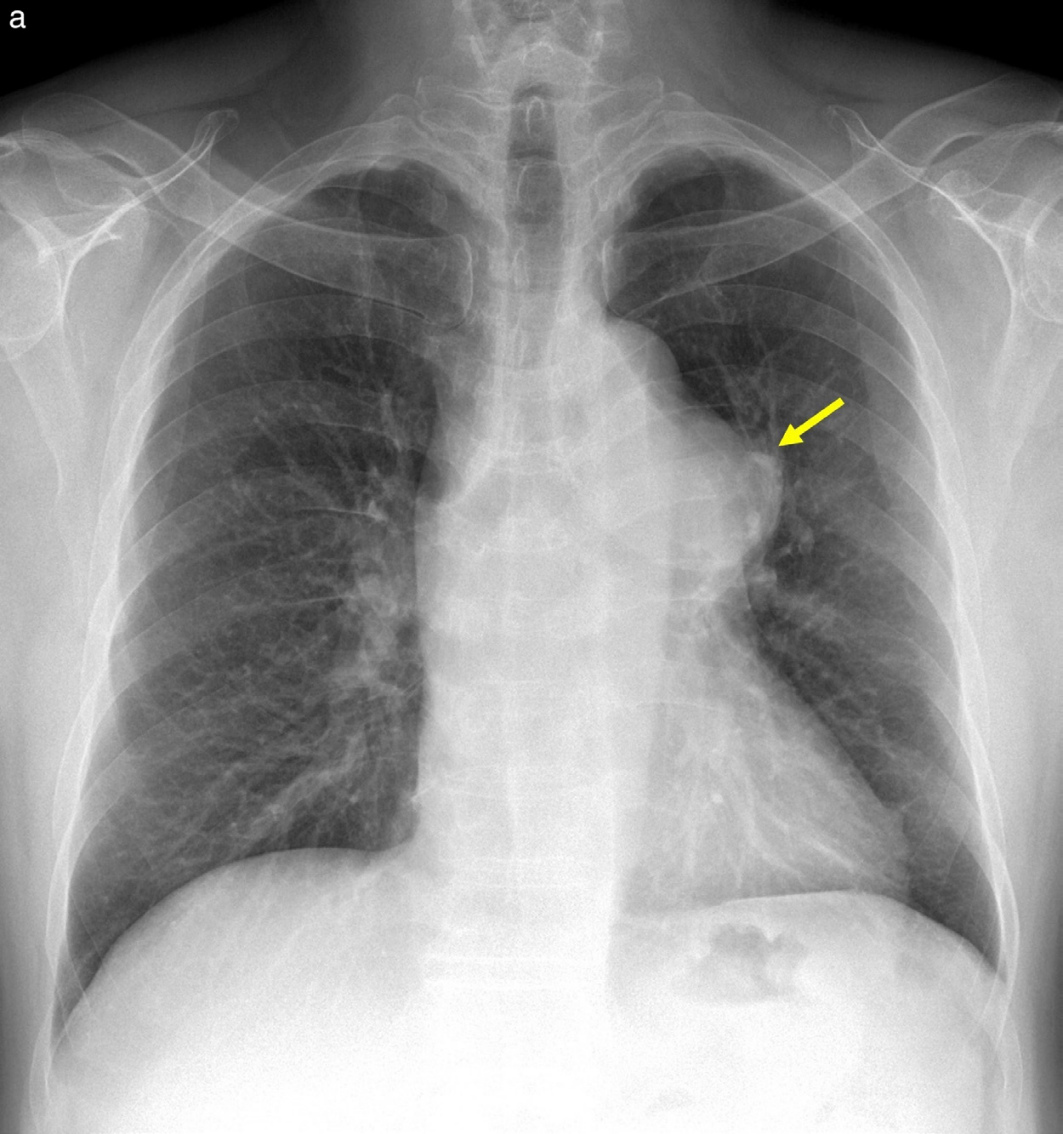

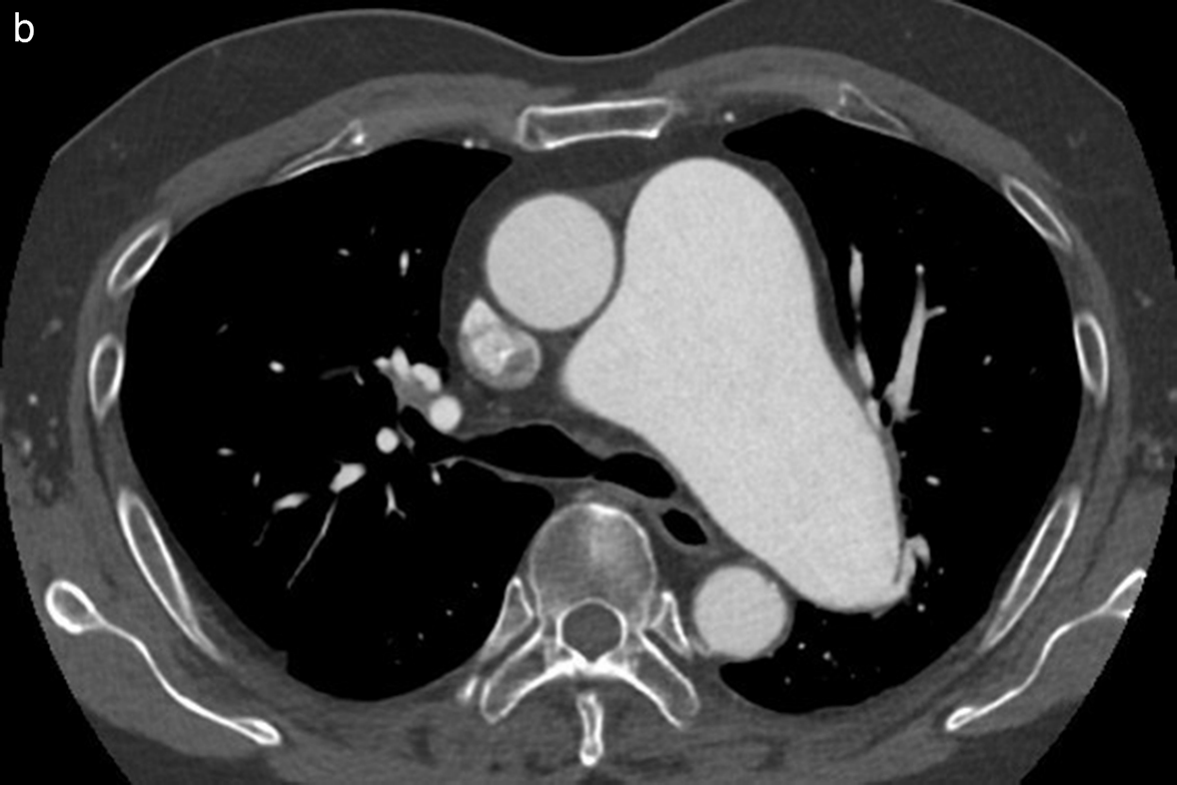

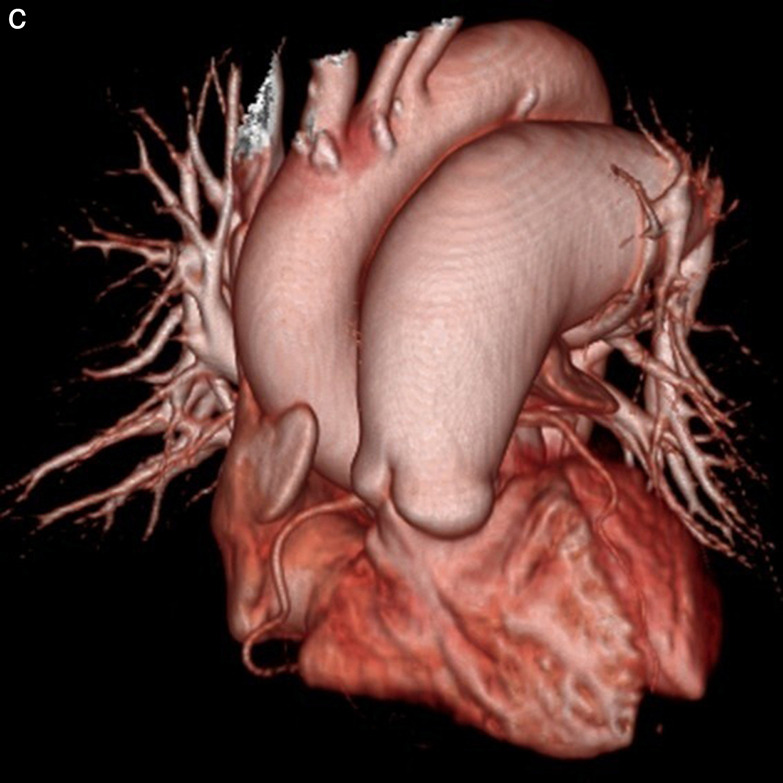
Idiopathic dilatation of the pulmonary trunk. **(a)** Chest radiography shows bulging of the left mediastinal border (arrow). **(b–c)** Axial CT **(b)** and volume‑rendered **(c)** images show marked dilatation of the pulmonary trunk and proximal pulmonary arteries.

Chest radiography may show bulging of the left mediastinal border, while contrast‑enhanced CT or MRI can confirm the abnormal enlargement of the main pulmonary trunk, with or without dilatation of both main pulmonary arteries (Figure 4) [11]. Although definite surgical guidelines are not available, surgery is typically suggested for symptomatic patients or when the pulmonary artery diameter exceeds 5 cm [9, 12].

### Proximal interruption of the pulmonary artery

Proximal interruption of the PA is a rare congenital anomaly characterized by the abrupt termination of the PA near the hilum. The affected lung is perfused via systemic collaterals—primarily bronchial arteries, with contributions from the intercostal, internal mammary, subclavian, and innominate arteries [13, 14]. These collateral vessels may become hypertrophied, predisposing patients to hemoptysis. Although some remain asymptomatic, most present during adolescence or early adulthood with recurrent infections, exertional dyspnea, or hemoptysis [15].

**Figure 5:**
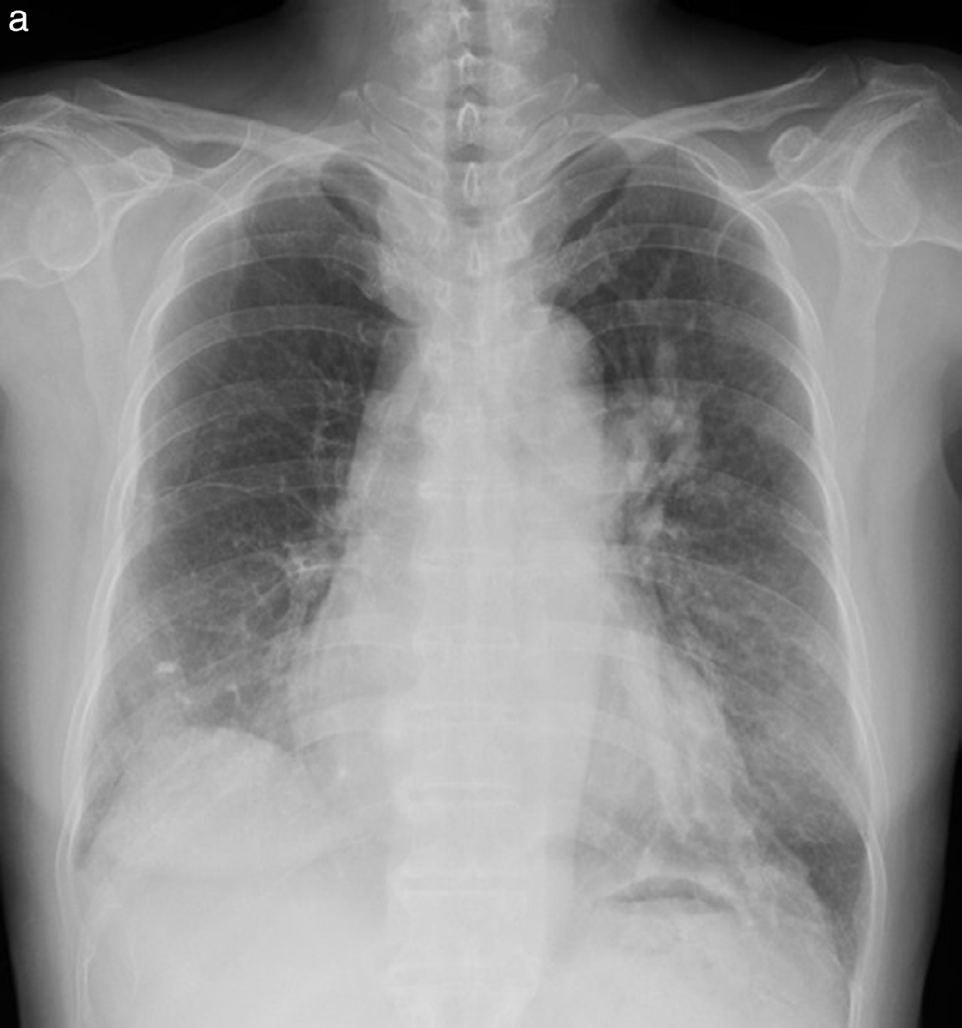

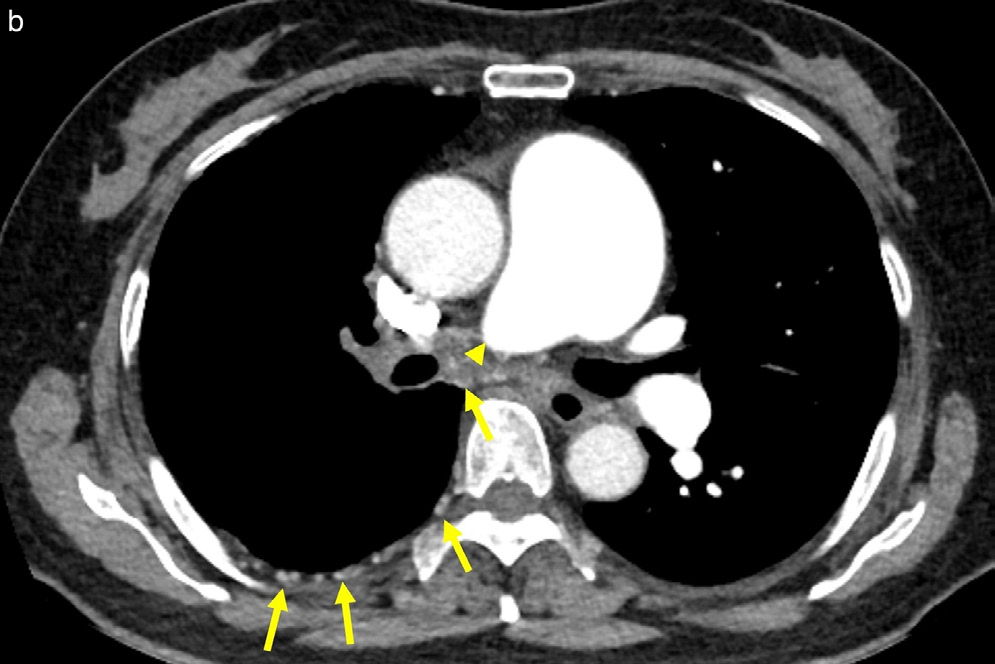

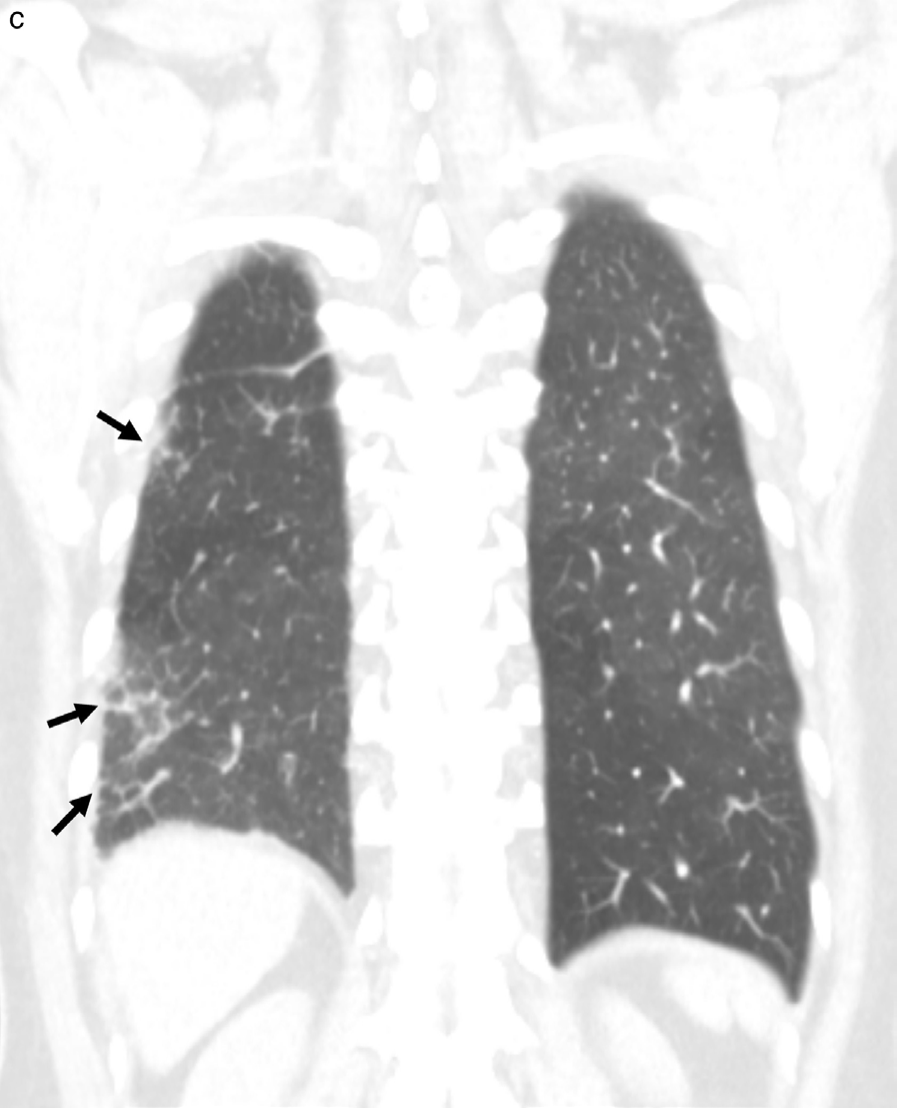

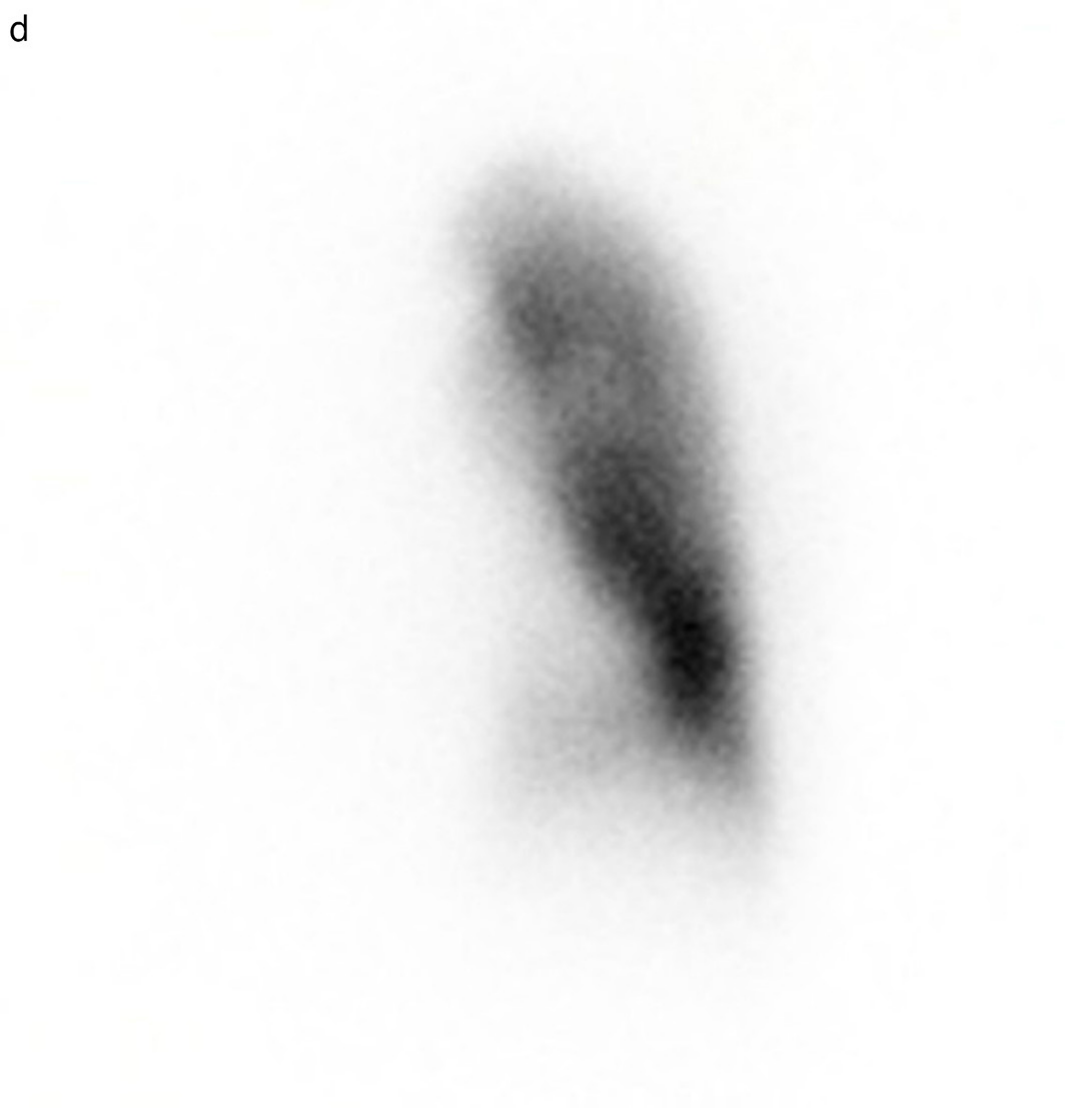
Proximal interruption of the right pulmonary artery. **(a)** Chest radiograph shows small right hilum. **(b)** Axial CT image demonstrates abrupt termination of the RPA (arrowhead) and collateral vessels (arrows). **(c)** A coronal, lung‑window CT image shows peripheral reticular opacities in the right lower lobe, corresponding to engorged subpleural collateral vessels (arrows). **(d)** Tc‑99 m MAA lung perfusion scan shows complete absence of perfusion to the right lung.

Chest radiographs of this condition typically show ipsilateral lung volume loss with a small hilum, mediastinal shift, and compensatory hyperinflation of the contralateral lung [15]. Curvilinear or reticular opacities in the subpleural lung may represent enlarged collateral vessels (Figure 5a) [13, 14].

CT allows for a definitive diagnosis by demonstrating the complete absence or termination of the affected PA within 1 cm of its origin. Collateral vessel hypertrophy can also be directly visualized. Additional findings may include serrated pleural thickening and parenchymal bands, reflecting transpleural collaterals bridging the systemic and peripheral pulmonary vessels [15]. Lung perfusion scintigraphy can demonstrate complete unilateral absence of perfusion, indicating systemic collateral supply to the affected lung (Figure 5b–5d) [16].

Treatment is not required if there are no signs of cardiopulmonary compromise. However, surgery is suggested for patients with life‑threatening infections or hemorrhages [17].

### Pulmonary stenosis

Pulmonary stenosis (PS), which is predominantly congenital, is most commonly of valvular origin (approximately 90% of cases), followed by subvalvular (infundibular) and supravalvular types [18]. Valvular PS is typically caused by a dome‑shaped pulmonary valve (PV) and, less commonly, dysplastic, bicuspid, and quadricuspid PV [19]. The severity of obstruction determines clinical presentation, which may range from incidental findings in mild PS to systemic venous congestion in moderate to severe PS [18].

**Figure 6:**
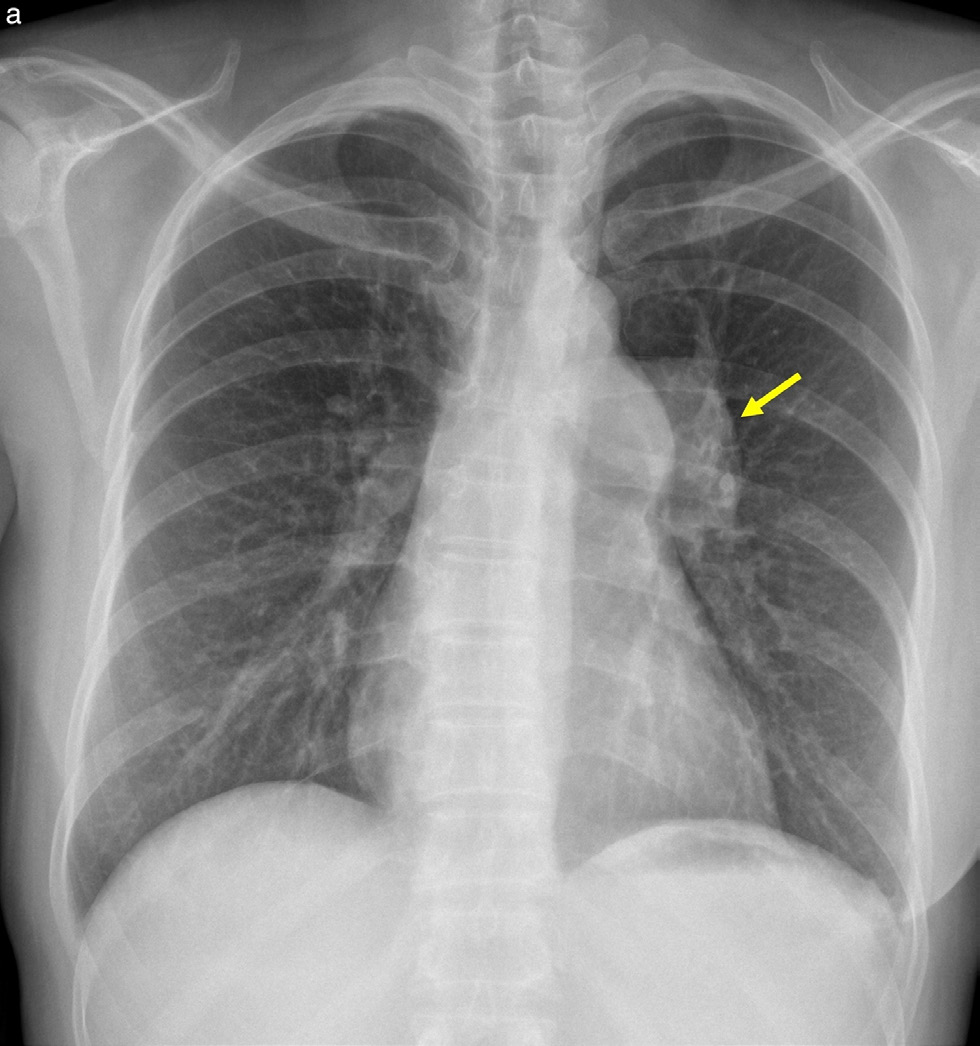

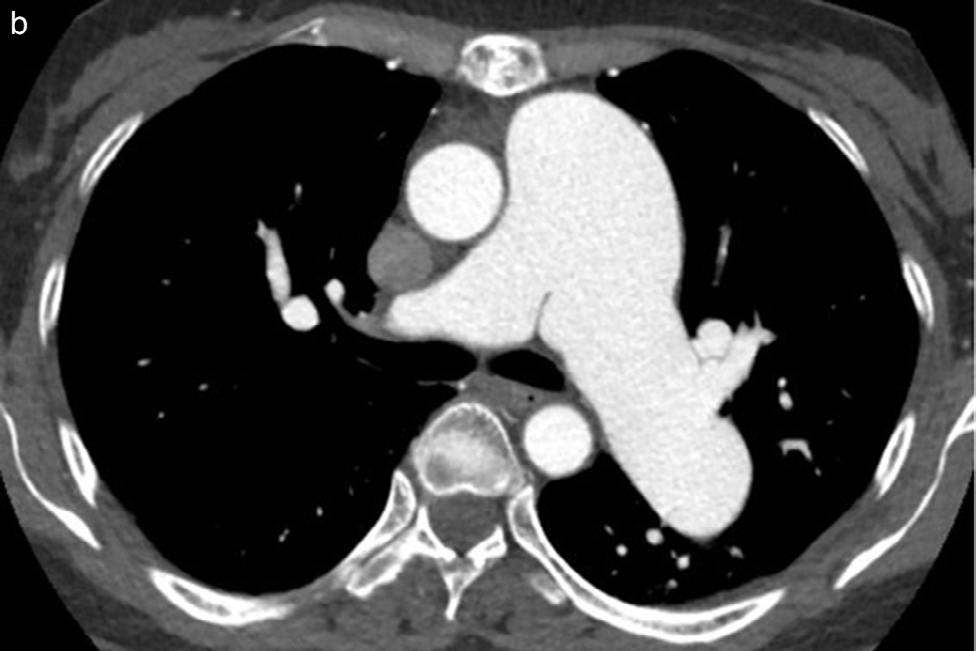

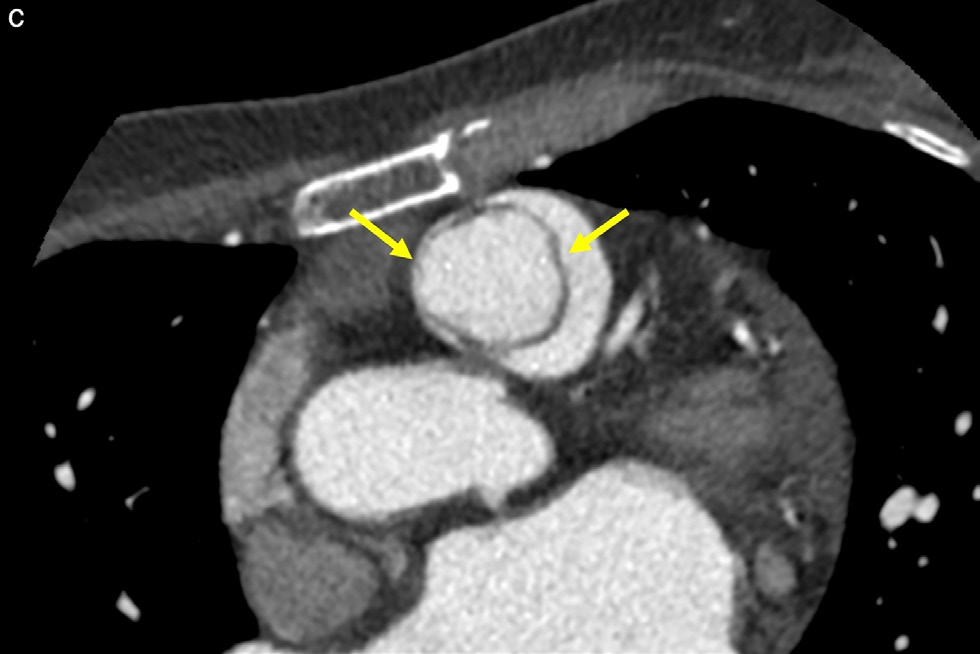

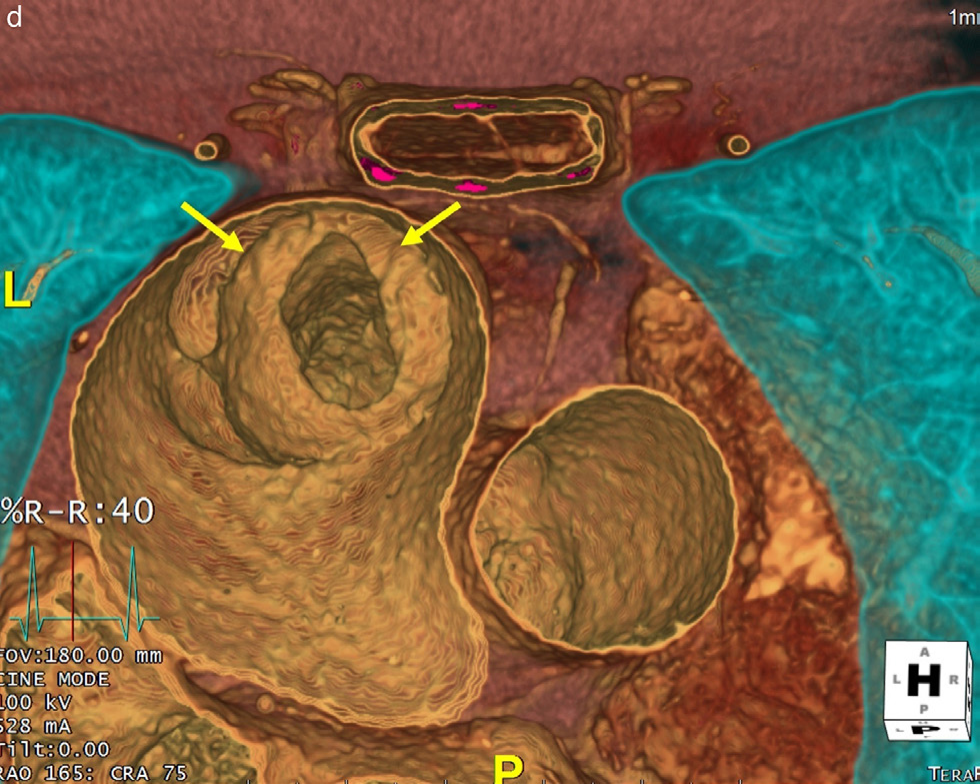
Valvular pulmonary stenosis. **(a)** Chest radiograph shows prominent left hila (arrow). **(b)** Axial CT image demonstrates dilatation of the main pulmonary artery and the left pulmonary artery. **(c–d)** Systolic‑phase ECG‑gated CT images show a stenotic bicuspid pulmonary valve (arrows) in en face **(c)** and volume‑rendered **(d)** views.

Chest radiography may demonstrate post‑stenotic dilatation of the main pulmonary artery and the LPA due to the direction of the high‑pressure blood flow across the stenotic valve, resulting in asymmetric hila or a prominent left mediastinal contour (Figure 6a). Contrast‑enhanced CT can depict this dilatation and may show secondary signs such as right ventricular hypertrophy or enlargement. Thickening and fusion of the valve leaflet, narrowing of the valve orifice, or bulging of the PV can be evaluated on ECG‑gated CT [18, 19] (Figure 6b–6d).

Balloon valvuloplasty is the treatment of choice in patients with hemodynamically significant valvular PS [11].

## Conclusion

Congenital anomalies of the pulmonary arteries, though rare, have significant clinical implications in thoracic imaging. Familiarity with the characteristic imaging features of pulmonary artery anomaly is essential for prompt diagnosis and appropriate clinical management.

## References

[r1] Zucker EJ. Cross‑sectional imaging of congenital pulmonary artery anomalies. Int J Cardiovasc Imaging. 2019;35(8):1535–1548. 10.1007/s10554-019-01643-4. 31175525

[r2] Sade RM, Rosenthal A, Fellows K, Castaneda AR. Pulmonary artery sling. J Thorac Cardiovasc Surg. 1975;69(3):333–346. 1117725

[r3] Capitanio MA, Ramos R, Kirkpatrick JA. Pulmonary sling: Roentgen observations. Am J Roentgenol Radium Ther Nucl Med. 1971;112(1):28–34. 10.2214/ajr.112.1.28. 5582030

[r4] Duong P, Mathur S, Miller OI. Partial anomalous left pulmonary artery. Eur Heart J Cardiovasc Imaging. 2018;19(2):237. 10.1093/ehjci/jex242. 29040408

[r5] Restrepo CS, Gonzalez TV, Baxi AJ, Saboo SS. Partial anomalous left pulmonary artery anterior versus posterior types: A systematic review. Tomography. 2022;8(4):1947–1958. 10.3390/tomography8040163. 36006061 PMC9416361

[r6] Maldjian PD, Adams KR. Partial anomalous left pulmonary artery sling in an adult. J Clin Imaging Sci. 2020;10:5. 10.25259/JCIS_4_2020. 32123619 PMC7049876

[r7] Sen S, Winlaw DS, Sholler GF. Partial anomalous left pulmonary artery: Report of two cases and review of literature. Cardiol Young. 2015;25(5):1012–1014. 10.1017/S1047951114001528. 25160653

[r8] Wang SY, Gao W, Zhong YM, et al. Multislice computed tomography assessment of tracheobronchial patterns in partial anomalous left pulmonary artery. J Comput Assist Tomogr. 2017;41(6):983–989. 10.1097/RCT.0000000000000623. 28448421

[r9] Agarwal S, Chowdhury UK, Saxena A, Ray R, Sharma S, Airan B. Isolated idiopathic pulmonary artery aneurysm. Asian Cardiovasc Thorac Ann. 2002;10(2):167–169. 10.1177/021849230201000219. 12079946

[r10] Serasli E, Antoniadou M, Steiropoulos P, et al. Low‑pressure pulmonary artery aneurysm presenting with pulmonary embolism: A case series. J Med Case Rep. 2011;5:163. 10.1186/1752-1947-5-163. 21518463 PMC3108942

[r11] Cortopassi IO, Gosangi B, Asch D, Bader AS, Gange CP, Rubinowitz AN. Diseases of the pulmonary arteries: Imaging appearances and pearls. Clin Imaging. 2022;91:111–125. 10.1016/j.clinimag.2022.08.018. 36067656

[r12] Kuwaki K, Morishita K, Sato H, Urita R, Abe T. Surgical repair of the pulmonary trunk aneurysm. Eur J Cardiothorac Surg. 2000;18(5):535–539. 10.1016/s1010-7940(00)00568-6. 11053813

[r13] Kieffer SA, Amplatz K, Anderson RC, Lillehei CW. Proximal interruption of a pulmonary artery. Am J Roentgenol Radium Ther Nucl Med. 1965;95(3):592–597. 10.2214/ajr.95.3.592. 5844925

[r14] Sherrick DW, Kincaid OW, Dushane JW. Agenesis of a main branch of the pulmonary artery. Am J Roentgenol Radium Ther Nucl Med. 1962;87:917–928. 13911733

[r15] Castaner E, Gallardo X, Rimola J, et al. Congenital and acquired pulmonary artery anomalies in the adult: Radiologic overview. Radiographics. 2006;26(2):349–371. 10.1148/rg.262055092. 16549603

[r16] Tsubamoto M, Fujita M, Okada A, et al. Isolated unilateral proximal interruption of the pulmonary artery: Findings of high‑resolution computed tomography and three‑dimensional volume rendering imaging of the pleura. Radiol Case Rep. 2017;12(1):19–24. 10.1016/j.radcr.2016.11.011. 28228870 PMC5310394

[r17] Ryu DS, Spirn PW, Trotman‑Dickenson B, et al. HRCT findings of proximal interruption of the right pulmonary artery. J Thorac Imaging. 2004;19(3):171–175. 10.1097/01.rti.0000130598.86945.b9. 15273613

[r18] Carter BW, Lichtenberger JP III, Wu CC. Congenital abnormalities of the pulmonary arteries in adults. AJR Am J Roentgenol. 2014;202(4):W308–W313. 10.2214/AJR.13.11759. 24660728

[r19] Saremi F, Gera A, Ho SY, Hijazi ZM, Sánchez‑Quintana D. CT and MR imaging of the pulmonary valve. Radiographics. 2014;34(1):51–71. 10.1148/rg.341135026. 24428282

